# Supplementary material for: Bridging organ transcriptomics for advancing multiple organ toxicity assessment with a generative AI approach
Source: NPJ Digit Med. 2024 Nov 5;7:310. doi: 10.1038/s41746-024-01317-z (PMC11538515; doi:10.1038/s41746-024-01317-z)
Supplement: Supplementary file 1 — Supplementary Information [file 41746_2024_1317_MOESM1_ESM.pdf]

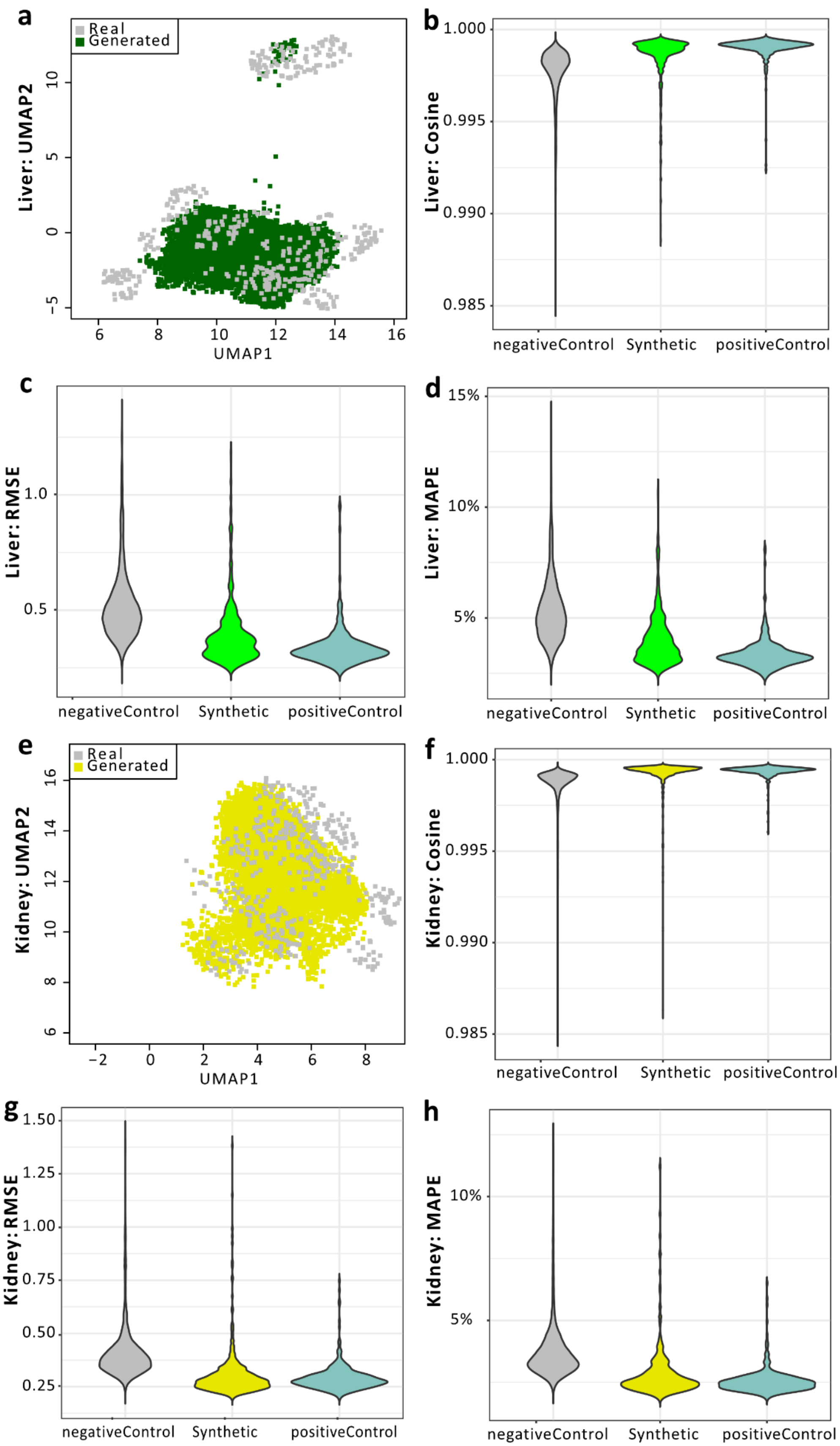

a
Real
Generated
Liver: UMAP2
UMAP1
−5
0
5
10
6
8
10
12
14
16
b
Liver: Cosine
1.000
0.995
0.990
0.985
negativeControl
Synthetic
positiveControl
c
Liver: RMSE
1.0
0.5
negativeControl
Synthetic
positiveControl
d
Liver: MAPE
15%
10%
5%
negativeControl
Synthetic
positiveControl
e
Real
Generated
Kidney: UMAP2
UMAP1
6
8
10
12
14
16
−2
0
2
4
6
8
f
Kidney: Cosine
1.000
0.995
0.990
0.985
negativeControl
Synthetic
positiveControl
g
Kidney: RMSE
1.50
1.25
1.00
0.75
0.50
0.25
negativeControl
Synthetic
positiveControl
h
Kidney: MAPE
10%
5%
negativeControl
Synthetic
positiveControl

Supplementary Figure 1: Performance of TransTox on the test TG-GATEs. Evaluation using UMAP, cosine similarity, RMSE, and MAPE for liver (**a** to **d**) and kidney (**e** to **h**). In negative control group, measurements such as cosine similarity were calculated between any two real transcriptomic profiles (excluding biological duplicates) within an organ. In positive control group, each measurement was calculated from the pairwise samples of biological replicates within an organ, providing a reference point for the achievable boundaries in an experiment.

Supplementary Data:

Supplementary Data 1: Important genes. Important genes are those with an absolute log2 fold change greater than 1 in at least one treatment condition. This table includes important genes from liver, kidney, and a combined set of liver and kidney.

Supplementary Data 2: DrugMatrix meta data. This table provides the DrugMatrix metadata, including compound name, dose, time, and other relevant details, for the liver and kidney transcriptomic profiles analyzed in this study.

Supplementary Data 3: TG-GATEs meta data and pathology findings. This table includes TG-GATEs metadata, such as compound name, dose, time, and other relevant details, for the liver and kidney transcriptomic profiles used in this study. Pathology findings associated with these treatments are also provided.
